# Association between severity of diabetic complications and risk of cancer in middle‐aged patients with type 2 diabetes

**DOI:** 10.1111/jdi.14364

**Published:** 2024-11-22

**Authors:** Yao‐Hsien Tseng, Yu‐Tse Tsan, Pau‐Chung Chen

**Affiliations:** ^1^ Department of Post‐Baccalaureate Medicine National Chung Hsing University Taichung Taiwan; ^2^ Division of Endocrinology and Metabolism Tungs' Taichung Metroharbor Hospital Taichung Taiwan; ^3^ Division of Occupational Medicine, Department of Emergency Medicine Taichung Veterans General Hospital Taichung Taiwan; ^4^ Department of Occupational Safety and Health Office Taichung Veterans General Hospital Taichung Taiwan; ^5^ School of Medicine Chung Shan Medical University Taichung Taiwan; ^6^ Institute of Environmental and Occupational Health Sciences National Taiwan University Taipei Taiwan; ^7^ Department of Public Health National Taiwan University Taipei Taiwan; ^8^ Department of Environmental and Occupational Medicine National Taiwan University Hospital Taipei Taiwan; ^9^ National Institute of Environmental Health Sciences National Health Research Institutes Miaoli Taiwan

**Keywords:** Cancer risk, Severity of diabetic complications, Type 2 diabetes mellitus

## Abstract

**Aim:**

Hyperglycemia was found to be associated with an increased risk of cancer in a general population cohort. However, it remains to be established whether the severity of diabetic complications is associated with cancer risk in patients with diabetes.

**Materials and Methods:**

We used the National Health Insurance Research Database from 2000 through 2013, including those with newly diagnosed diabetic patients (*n* = 616,742). We collected all vascular and metabolic complications to develop an adapted diabetic complication severity index (aDCSI), ranging from 0 to 13 annually, as proxies of the severity of diabetic complications and performed follow‐up from the onset of diabetes until incident cancer, death, or the study end.

**Results:**

Within the mean follow‐up period of 9 years, the rates of cancer incidence per 100,000 person‐years were 815.2 vs 482.0 and 611.1 vs 358.9 for the top vs bottom quartiles, respectively, of aDCSI in men and women (adjusted HRs 1.17 (95% CI 1.10–1.25) and 1.20 (95% CI 1.10–1.30), respectively). The risk of cancer was 1.7‐ to 1.9‐fold for the top vs bottom quartiles of aDCSI in diabetic onset age of 40–44 (HRs 1.74 (95% CI, 1.39–2.18) in men and HRs 1.93 (95% CI, 1.39–2.66) in women). However, among patients with diabetic onset age of 60–64, the associations between the severity of diabetic complications and cancer risk were attenuated.

**Conclusions:**

Patients with higher severity of diabetic complications have an increased risk of cancer compared to those with the lowest severity, particularly for those with earlier onset and greater severity of diabetic complications.

## Graphical abstract

This study investigates the link between the severity of diabetic complications and cancer risk using data from the National Health Insurance Research Database (2000–2013) involving 616,742 newly diagnosed diabetic patients. Results indicate that higher severity of diabetic complications significantly increases cancer risk, particularly in patients with early‐onset diabetes, with adjusted hazard ratios of 1.17 for men and 1.20 for women in the highest severity quartile compared to the lowest. The association was strongest in patients aged 40–44 at diabetes onset but attenuated in those aged 60–64.

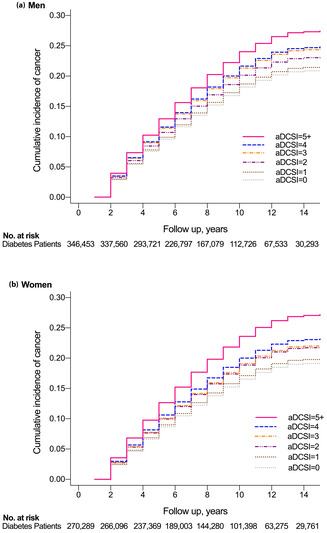

## INTRODUCTION

The global prevalence of diabetes is rising, leading to substantial health and economic burdens^1^. Many patients present with chronic complications, including both vascular and nonvascular issues, which significantly increase both direct medical costs and indirect economic burdens^2^. Projections indicate that by 2045, approximately 11.9% of the global adult population will have diabetes, further exacerbating these challenges^3^.

In addition to the economic and health burdens posed by diabetes, the condition is also associated with an increased risk of certain cancers, particularly liver, pancreatic, and breast cancers^4^, ^5^, ^6^. Moreover, high blood sugar levels have been shown to elevate cancer risk even in individuals without diabetes or prediabetes^7^, ^8^. While intensive glycemic control has been shown to reduce microvascular complications, its effect on cancer risk remains inconsistent^9^.

Several biological mechanisms may explain these increased cancer risks. The accumulation of advanced glycation end products (AGEs) and oxidative stress are central to the progression of diabetic complications and may also promote cancer development^10^. Elevated levels of AGEs drive both microvascular and macrovascular complications in diabetes while simultaneously activating pathways that heighten cancer risk^11^. These mechanisms underscore the need for a clearer understanding of the link between the severity of diabetic complications and cancer risk, not only for individuals with high glucose levels but also for those experiencing significant diabetic complications.

Here, we define seven diabetic complications of metabolic, microvascular, and macrovascular disease, each graded as 0, 1, or 2, with a total range of 0–13, as proxies for the severity of diabetic complications. Our study aimed to assess the association between the degree of disease severity and the risk of cancer in patients with diabetes.

## METHODS

We used data from the Taiwan National Health Insurance Research Database (NHIRD), which provides compulsory health insurance to 23 million people in Taiwan. The cohort with diabetes was identified from the NHIRD, and information on all medical behaviors and treatments in the population with diabetes was included^12^. The ethics review board of the National Taiwan University College of Public Health approved the present study. This study strictly adhered to the confidentiality guidelines and regulations for protecting personal electronic data^13^. The reimbursement data maintained by Taiwan's National Health Research Institute used in this study comprise anonymized files suitable for research.

### Study participants

We conducted a 13‐year retrospective cohort study between 2000 and 2013 that involved patients newly diagnosed with diabetes and for whom the diagnosis had been validated with 90% sensitivity and 90% positive predictive value^14^. To ensure that the cohort population comprised people with diabetes exclusively, subjects who had not received any diabetic medication during the entire study period were excluded. The exclusion criteria included the following groups: those diagnosed with diabetes in the first year (to ensure new onset diabetes) or the last year of the operation of the database (to ensure that the follow‐up duration was at least 1 year); missing age or sex data; younger than 40 or older than 65 years; diagnosed with any malignant neoplasm (ICD‐9 codes 140‐208) prior to the initial ambulatory care visit that resulted in the diabetes diagnosis.

### Diabetes Complication Severity Index

The Diabetes Complications Severity Index (DCSI) was developed by Young using a clinical database to model the severity of the disease^15^. The DCSI includes seven categories of complications: cardiovascular disease, stroke, peripheral vascular disease, retinopathy, nephropathy, neuropathy, and metabolism, with a total score of 0–13 (Appendix S1). The performance of the DCSI in predicting mortality and risk of hospitalization is well accepted^15^. The adapted DCSI (aDCSI) was created from a claims database and was validated in the Taiwan NHIRD, reporting a good performance; it shows an ability to predict hospitalization similar to that of the DCSI^16^, ^17^. We created an annual mean aDCSI to model the severity of diabetic complications as a time‐dependent variable. Categories of aDCSI scores from 0 to 5+ were made, whereby the categories of aDCSI scores above five were combined due to the small sample sizes.

### Outcome definitions

All cancers from any cause were outcomes of interest, defined as ICD‐9 codes 140–208. Specific cancers were also analyzed in subgroup analysis, including colorectal cancer (ICD‐9 153–154), hepatocellular cancer (ICD‐9 155), pancreatic cancer (ICD‐9 157), breast cancer (ICD‐9 174), prostate cancer (ICD‐9 185), stomach cancer (ICD‐9 151), kidney cancer (ICD‐9 189.0), urinary tract cancer (ICD‐9 189.1 and 189.2), bladder cancer (ICD‐9 188), lung cancer (ICD‐9 162), ovarian cancer (ICD‐9 183), endometrial cancer (ICD‐9 182), lymphoma (ICD‐9 200–202.28, 202.7–202.98), and leukemia (ICD‐9 204–208).

We included only patients with a cancer diagnosis in the Registry of Catastrophic Illness Database, which ensures high accuracy and validity *via* the rigorous approval process reviewed by specialists. To ensure sufficient observation time, cancer was defined as the first occurrence of cancer during the follow‐up period from at least 1 year after the diagnosis of diabetes to death or December 31, 2013. For subgroup analysis, the lag time was set to 1, 2, and 3 years after enrollment. The study began with follow‐up after the onset of diabetes and ended with the following censoring events: diagnosis of cancer, death, or the end of the study, whichever occurred first. Subgroup analysis based on the duration of diabetes was performed to determine the association of the severity of diabetic complications with incident cancer according to the duration of diabetes (<6 and ≥6 years).

All potential confounders related to cancer were identified during follow‐up, including the baseline covariates of sex, income, and level of urbanization, and the following time‐varying covariates that change annually: age; status of hypertension and hyperlipidemia; medication for diabetes, hypertension, and hyperlipidemia; and use of aspirin. The status of hypertension and hyperlipidemia in each year was defined as methods in diabetic definition with ICD‐9 codes 401–405 and 272, respectively. To define the use of medication, we calculated the defined daily dose above 28 in each year. Urbanization was divided into four categories, with level 1 referring to the most urbanized communities and level 4 referring to the least urbanized communities (Appendix S1).

### Statistical analysis

The parameters of age, income, and urbanization were calculated as means or percentages. Age, comorbid disease status, use of medication, and aDCSI score were time‐dependent variables, which varied per year. We categorized diabetes onset into two groups for clarity in assessing cancer risk: ‘earlier‐onset’ diabetes, defined as diagnosis between the ages of 40–50 years, and ‘late‐onset’ diabetes, defined as diagnosis at ages 60–64 years. To evaluate the estimates of cancer risk, the aDCSI score was treated as a continuous independent variable or a categorical variable from 0 to ≥5 and was compared with the aDCSI score at the lowest stratum as a reference.

A competing risk model accounting for competing causes of death incorporated time‐dependent covariates using the Cox proportional hazard model to estimate the hazard ratios (HRs) and 95% confidence intervals (CIs) associated with the aDCSI scores^18^. With regard to the analysis of specific types of cancer, two competing risks of death or another cancer that was not an outcome of interest were considered in the Cox survival analysis. Estimates of the cumulative incidence of cancer from any cause were plotted stratified by the degree of aDCSI from 0 to 5+. We stratified the breast cancer analysis into two groups, age <50 years and age ≥50 years, as a surrogate for menopausal status. We evaluated crude and adjusted Cox proportional hazard models to control for potential confounding factors, including age and socioeconomic status and further adjusted the model with the comorbid disease according to the status of hypertension and hyperlipidemia as well as medication use. Alternatively, we conduct a nested case–control analysis using the same data of the cohort with incidence density sampling at a 1:4 ratio and match on the covariates of gender, status of income urbanization, and age (±2 years). We used conditional logistic regression to analyze the association of time‐varying aDCSI with the risk of cancer, which was displayed as odds ratios and 95% confidence intervals.

Subgroup analyses were performed according to age at diabetes onset (5‐year intervals), diabetes duration (<6 or ≥6 years), and lag time (1, 2, or 3 years) of follow‐up. All analyses were stratified by sex. Statistical analyses were performed using SAS version 9.4 (SAS Institute Inc., Cary, NC). Statistics tests were 2‐sided, and a *P* value less than 0.05 was regarded as statistically significant.

## RESULTS

### Baseline characteristics of patients with diabetes

**Figure 1:**
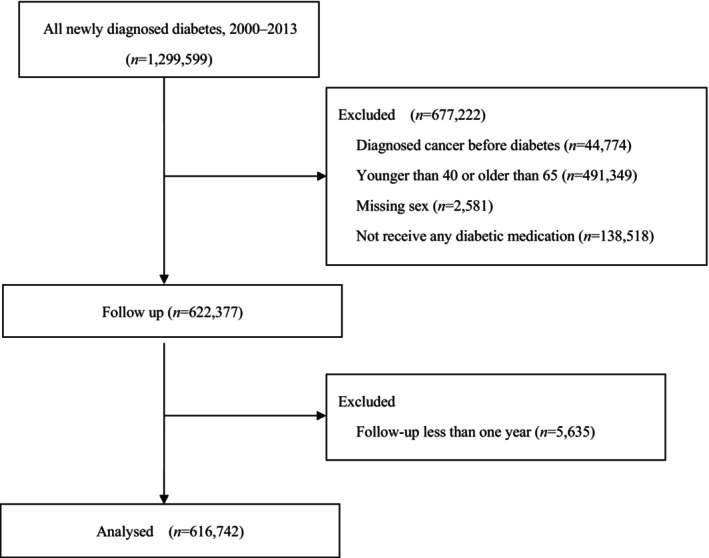
Flow diagram.

Among the 1,299,599 patients with diabetes initially included in this cohort from 2000 through 2013, we excluded those diagnosed with cancer before the onset of diabetes (*n* = 44,774), those younger than 40 or older than 65 years (*n* = 491,349), those with missing data for sex and socioeconomic status (*n* = 2,581), those who did not receive any diabetic medication treatment during follow‐up (*n* = 138,518) and those followed up less than 1 year (*n* = 5,635); the remaining 616,742 diabetes patients were retained (Figure 1). The baseline characteristics of the patients are shown in Table 1 according to sex. The mean (±SD) age at baseline was 53.0 ± 6.6 years, 43.8% were women, and the median follow‐up period was 9 years.

**Table 1:** Baseline characteristics of patients with type 2 diabetes

	Men (*n* = 346,453)	Women (*n* = 270,289)
Variable		
Age—years^†^	52.3 ± 6.7	53.9 ± 6.5
Follow‐up—years^†^	8.6 ± 3.8	9.2 ± 3.8
Income (NTD)—No. (%)		
0	18,612 (5.4)	45,030 (16.7)
1–15,840	50,270 (14.5)	36,780 (13.6)
15,840–25,000	151,075 (43.6)	153,005 (56.6)
>25,000	126,496 (36.5)	35,474 (13.1)
Urbanization—No. (%)		
Low	153,288 (44.2)	113,545 (42)
Moderate	140,356 (40.5)	111,437 (41.2)
High	36,115 (10.4)	30,167 (11.2)
Very high	16,694 (4.8)	15,140 (5.6)
Medical diseases—No. (%)		
Hypertension	85,972 (24.8)	81,744 (30.2)
Hyperlipidemia	58,714 (17.0)	45,152 (16.7)
Medication—No. (%)		
Metformin	14,636 (4.2)	8,810 (3.3)
Sulfonylurea	30,772 (8.9)	18,928 (7.0)
Acarbose	980 (0.3)	641 (0.2)
TZD	1,278 (0.4)	713 (0.3)
DPP4 inhibitor	197 (0.06)	96 (0.04)
Insulin	3,975 (1.2)	1,721 (0.6)
CCB	8,497 (2.5)	9,045 (3.4)
Beta‐blocker	20,498 (5.9)	23,015 (8.5)
ACEI	29,984 (8.7)	25,619 (9.5)
ARB	22,044 (6.4)	17,681 (6.5)
Aspirin	28,319 (8.2)	20,100 (7.4)
Statins	16,373 (4.7)	14,491 (5.4)
aDCSI^†^	0.36 ± 0.84	0.39 ± 0.81

^†^ Values represent means ± SD.

ACEI, angiotensin‐converting enzyme inhibitor; aDCSI, adapted Diabetes Complications Severity Index; ARB, angiotensin II receptor blocker; CCB, calcium channel blocker; DPP4, dipeptidyl peptidase 4; NTD, New Taiwan dollars; TZD, thiazolidinedione.

### The severity of diabetic complications and cancer risk

**Figure 2:**
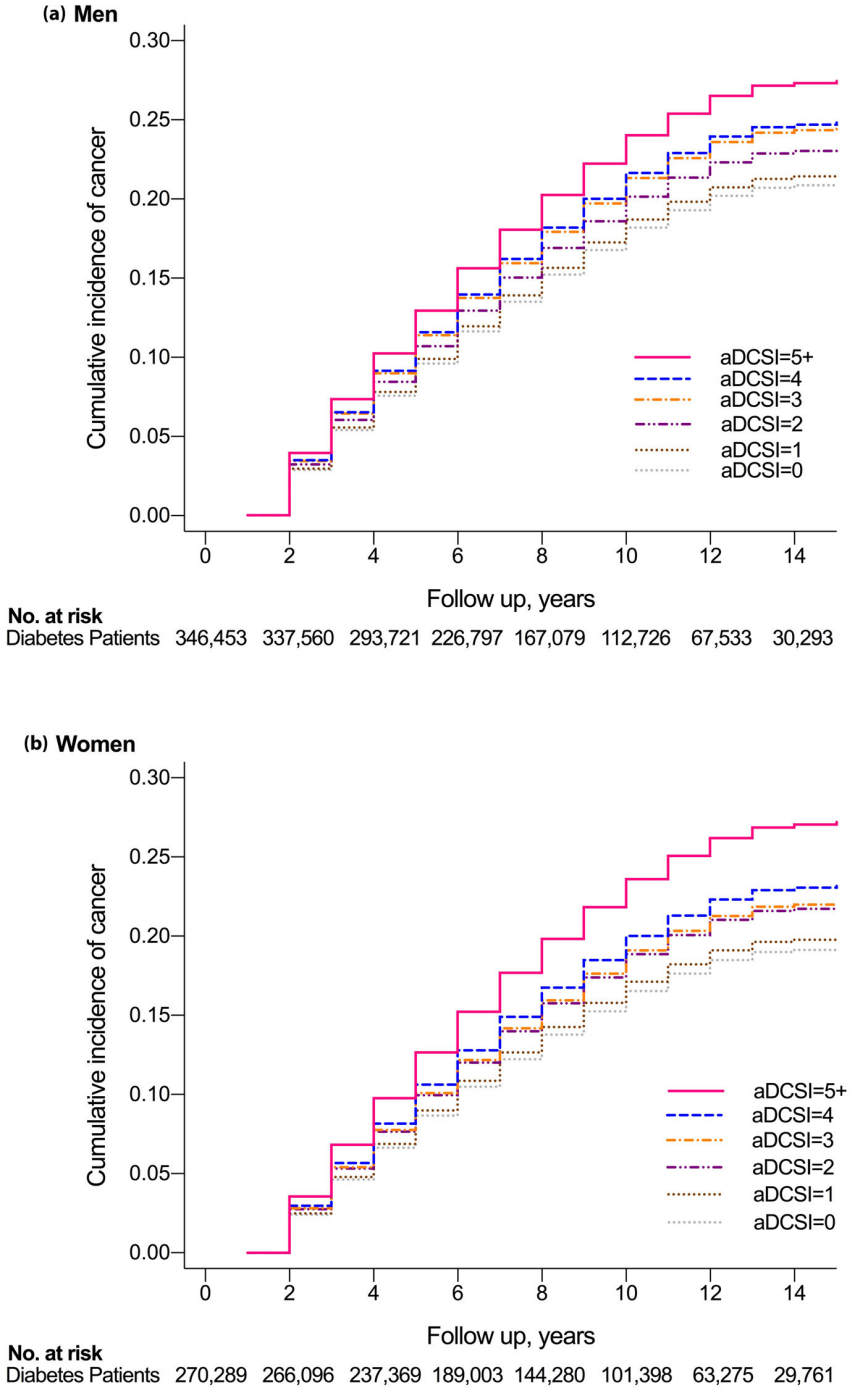
Cumulative incidence curve of cancers from any cause according to the aDCSI. Cumulative incidence rates for cancers from any cause according to the aDCSI from 0 to 5+, as adjusted for age, sex and socioeconomic status accounting for the competing risk of death. (a) Males; (b) females. aDCSI, adapted Diabetes Complication Severity Index; CI, confidence interval.

Table 2 shows the HRs and incidence of cancer from any cause. The cumulative incidence curves of cancer from any cause according to an aDCSI score from 0 to 5+ are shown in Figure 2. Overall, the incidences of cancer from any cause per 100,000 person‐years were 482.0, 585.4, 662.1, 724.4, 748.5, and 815.2; and 358.9, 436.4, 501.3, 515.6, 544.2, and 611.1 among male and female patients with aDCSI scores of 0, 1, 2, 3, 4 and 5+, respectively. According to Cox regression analysis, the HRs for cancer from any cause in diabetes patients with the highest aDCSI scores vs those with the lowest aDCSI scores were 1.09 (95% CI, 1.08–1.10) and 1.09 (95% CI, 1.08–1.11) for men and women, respectively. The HRs for the same comparison were 1.05 (95% CI, 1.05–1.06) and 1.07 (95% CI, 1.05–1.08) with further adjustment for age, urbanization level, and income, respectively, and 1.03 (95% CI, 1.02–1.04) and 1.04 (95% CI, 1.02–1.05) with adjustment for coexisting illness and medication use, respectively. The increase in HRs for cancer from any cause associated with aDCSI scores of 1–5+ vs the reference increased substantially from 8.0% to 17% and from 7.0% to 20% in men and women, respectively. The nested case–control analysis comprised 27,934 cases with cancers and 111,736 matched controls. The estimates from nested case–control analysis were similar to those of the cohort study analysis (Table S1).

**Table 2:** Unadjusted and adjusted HRs for cancer according to the adapted Diabetes Complications Severity Index in quartiles

aDCSI	0	1	2	3	4	5+	*P* for trend
Men							
No. of cancers from any cause	6,992	3,542	2,995	1,550	997	1,192	
No. of cancers per 10^5^ person‐years (95% CI)	482.0 (481.2–482.7)	585.4 (583.9–586.9)	662.1 (660.2–664.1)	724.4 (721.3–727.5)	748.5 (744.4–752.5)	815.2 (811.0–819.4)	
Crude HR (95% CI)	1.00	1.07 (1.03–1.12)	1.23 (1.18–1.28)	1.35 (1.28–1.43)	1.42 (1.33–1.52)	1.63 (1.53–1.74)	<0.001
Adjusted HR^†^ (95% CI)	1.00	1.03 (0.99–1.08)	1.12 (1.08–1.17)	1.20 (1.13–1.26)	1.22 (1.14–1.31)	1.38 (1.29–1.47)	<0.001
Adjusted HR^‡^ (95% CI)	1.00	1.08 (1.04–1.12)	1.14 (1.09–1.19)	1.19 (1.13–1.26)	1.16 (1.09–1.24)	1.17 (1.10–1.25)	<0.001
Women							
No. of cancers from any cause	3,924	2,564	1,917	1,001	591	669	
No. of cancers per 10^5^ person‐years (95% CI)	358.9 (358.2–359.5)	436.4 (435.2–437.5)	501.3 (499.7–502.9)	515.6 (513.3–517.9)	544.2 (541.0–547.5)	611.1 (607.5–614.7)	
Crude HR (95% CI)	1.00	1.08 (1.03–1.14)	1.26 (1.19–1.33)	1.31 (1.22–1.40)	1.42 (1.30–1.55)	1.71 (1.58–1.87)	<0.001
Adjusted HR^†^ (95% CI)	1.00	1.04 (0.99–1.10)	1.16 (1.10–1.22)	1.17 (1.09–1.26)	1.24 (1.14–1.36)	1.49 (1.37–1.62)	<0.001
Adjusted HR^‡^ (95% CI)	1.00	1.07 (1.02–1.13)	1.15 (1.09–1.22)	1.15 (1.07–1.23)	1.15 (1.05–1.26)	1.20 (1.10–1.30)	<0.001

^†^ Adjusted for age, urbanization, and income.

^‡^ Adjusted for age, urbanization, income, and status of hypertension, hyperlipidemia, medication for hyperglycemia, hypertension, and hyperlipidemia and use of aspirin.

aDCSI, adapted Diabetes Complications Severity Index; CI, confidence interval; HR, hazard ratio.

Regarding the analysis stratified by baseline diabetes age of onset, the risk of cancer increased consistently with rising aDCSI scores in both women and men of onset ages in the 40–59‐year‐old categories (Table 3). Among those with a diabetes onset age of 40–44 years at the highest stratum of the aDCSI, the risk of cancer was approximately 1.7‐ to 1.9‐fold higher among those in the lowest aDCSI score stratum (HR for cancer from any cause, 1.74 (95% CI, 1.39–2.18) in men and 1.93 (95% CI, 1.39–2.66) in women). However, among patients with diabetes onset at ages of 60–64 years at the highest stratum of the aDCSI, the risk of cancer was approximately 1.3‐fold higher than in the corresponding reference for women compared to those with the lowest aDCSI scores. No significant association in men was found (HR for cancer from any cause, 1.05 (95% CI, 0.93–1.18) in men and 1.33 (95% CI, 1.16–1.54) in women).

**Table 3:** Adjusted HRs for cancer from any cause among patients with type 2 diabetes according to time‐dependent aDCSI scores and stratified according to baseline age^†^

Variable	Cancer from any cause (men)					Cancer from any cause (women)				
	40–44 years	45–49 years	50–54 years	55–59 years	60–64 years	40–44 years	45–49 years	50–54 years	55–59 years	60–64 years
aDCSI										
0	1.00	1.00	1.00	1.00	1.00	1.00	1.00	1.00	1.00	1.00
1	1.01 (0.88–1.15)	1.00 (0.91–1.10)	1.10 (1.01–1.19)	0.96 (0.88–1.04)	0.99 (0.91–1.08)	1.15 (0.96–1.38)	1.06 (0.93–1.21)	1.09 (0.99–1.21)	1.04 (0.94–1.15)	0.96 (0.88–1.06)
2	1.18 (1.02–1.36)	1.23 (1.11–1.36)	1.15 (1.05–1.27)	1.02 (0.94–1.11)	0.97 (0.89–1.05)	1.31 (1.06–1.61)	1.29 (1.11–1.49)	1.14 (1.02–1.29)	1.20 (1.08–1.34)	1.05 (0.95–1.16)
3	1.25 (1.02–1.53)	1.23 (1.07–1.42)	1.27 (1.12–1.43)	1.05 (0.94–1.18)	1.05 (0.94–1.16)	1.09 (0.79–1.50)	1.15 (0.93–1.41)	1.26 (1.08–1.47)	1.17 (1.02–1.35)	1.13 (1.00–1.28)
4	1.36 (1.05–1.76)	1.43 (1.21–1.69)	1.11 (0.95–1.30)	1.13 (0.99–1.29)	1.04 (0.92–1.17)	1.11 (0.74–1.67)	1.46 (1.14–1.88)	1.35 (1.11–1.65)	1.23 (1.03–1.46)	1.15 (0.99–1.33)
5+	1.74 (1.39–2.18)	1.68 (1.45–1.96)	1.49 (1.30–1.71)	1.20 (1.06–1.37)	1.05 (0.93–1.18)	1.93 (1.39–2.66)	1.89 (1.50–2.36)	1.68 (1.38–2.03)	1.32 (1.10–1.57)	1.33 (1.16–1.54)
Combined	1.09 (1.05–1.12)	1.09 (1.07–1.11)	1.06 (1.04–1.08)	1.03 (1.01–1.05)	1.01 (0.99–1.03)	1.10 (1.05–1.15)	1.10 (1.07–1.14)	1.08 (1.05–1.11)	1.05 (1.03–1.08)	1.05 (1.03–1.07)
P for trend	<0.001	<0.001	<0.001	0.002	0.267	<0.001	<0.001	<0.001	<0.001	<0.001

^†^ Adjusted for age, urbanization and income.

Abbreviations: aDCSI, adapted Diabetes Complications Severity Index; CI, confidence interval; HR, hazard ratio.

The HRs and incidence of cancer stratified by specific origin are shown in Table S2. The risk of stomach, pancreas, colorectal, prostate, lung, kidney, urinary, and bladder cancers and leukemia in men was significantly associated with a high aDCSI score, with HRs of 1.04–1.21. In female patients, hepatocellular, colorectal, kidney, urinary tract, and bladder cancers and leukemia were associated with high aDCSI scores with HRs of 1.05–1.30. However, the risk of postmenopausal breast cancer was inversely associated with aDCSI scores.

Moreover, subgroup analysis stratified by the duration of diabetes (<6 years or ≥6 years) revealed the same results for the two groups (Figure S1). Additionally, analysis of the lag time (≥1, ≥2, or ≥3 years) between the onset of diabetes and the development of cancer showed no effect on the direction of the HR for cancer (Figure S2).

## DISCUSSION

This study is the first to address the association between the severity of diabetic complications and the risk of cancer exclusively among patients with diabetes, filling a significant gap in understanding how these complications contribute to increased cancer risk. This study confirmed an 8–17% and 7–20% increase in the risk of cancers with higher aDCSI in males and females, respectively.

Evaluating the risk of cancer using glucose levels, which vary individually and change over time, biases the estimates of long‐term cancer outcomes. Indeed, total glycemic exposure based on the HbA1c level with the duration of exposure explains only ~11% of the risk of progression of diabetic complications^19^; furthermore, other factors not related to glucose levels, including blood glucose excursion, also contribute to complication risk^20^. Compared to conventional glucose control, lower levels of HbA1c due to intensive glucose control result in fewer diabetic complications but similar overall cancer incidence^9^, ^21^. In the ADVANCE trial, patients with severe hypoglycemia, who might have overlapping phenotypes associated with cancer^22^, were more likely to develop cancer than those without severe hypoglycemia^22^, ^23^.

To our best knowledge, there were no studies evaluating hyperglycemia and risk of cancer exclusively in patients with diabetes. Two population‐based studies were derived from health surveys or medical examinations, and potential confounders were not considered in assessing associations between hyperglycemia and incident cancer. A prospective cohort study of health insurance in Korea showed that patients of both genders with the highest levels of fasting glucose had higher incidence rates of cancer from any cause than those with the lowest concentrations of fasting glucose^8^. A population‐based field screening study in Sweden showed increased glucose levels to be associated with an overall increased risk of cancer in women but not in men^7^.

The studies compared cancer risk in patients, with fewer than 10% of whom had diabetes or prediabetes, with subjects with normal glucose levels as the reference. The HRs for cancer from any cause in these two studies were similar to ours, which were slightly attenuated when the population was limited to patients with diabetes.

The prevalence of obesity significantly increases with age^24^, and the effect of increased body mass index on subsequent postmenopausal breast cancer risk is relevant in patients with diabetes^25^, ^26^.

Women's Health Initiative clinical trials have identified the dose–response effect of obesity on the risk of postmenopausal breast cancer^27^. Overall, adiposity contributes to the risk of breast cancer more than does diabetes^28^, which may explain the inverse association between postmenopausal breast cancer risk and severity of diabetic complications in our study. Nonetheless, a higher incidence of kidney cancer was observed in diabetic women than in non‐diabetic women, with an increased HR of 1.6 that reached as high as 4.13 in patients with more comorbidities than in those with no comorbidities^29^. The risk of kidney cancer in our study was also markedly increased and was as high as 3.4–3.7 for the top vs bottom quartiles of the aDCSI in patients with diabetes. Compared to non‐diabetic men, several confounding factors for diabetic men have been observed, with controversial results in terms of the risk of prostate cancer^6^, ^30^. Our study, which was limited to subjects with diabetes, showed that the risk of prostate cancer was significantly associated with aDCSI scores.

In our study, associations between aDCSI scores and cancer from any cause decreased with increasing age of diabetes onset, with the highest HRs among patients with onset age of 40–50 years (earlier‐onset) in both sexes, whereas the risk of cancer was found to be attenuated in the stratum of onset age of 60–64 years (later‐onset age). Our study revealed a stronger association between higher aDCSI scores and cancer risk among individuals diagnosed with diabetes at a younger age (40–50 years), indicating that earlier‐onset diabetes may represent a critical window for increased cancer vulnerability.

In our study, we observed a differential association between aDCSI scores and cancer risk depending on the age at diabetes onset. Specifically, the highest HRs for cancer were seen in patients diagnosed with diabetes between the ages of 40 and 50 years, which we define as earlier‐onset diabetes. This association was most pronounced in both male and female patients. Conversely, we noted a reduced cancer risk among patients diagnosed at ages 60 and 64 years, referred to as later‐onset diabetes. The higher mortality rate from other causes in those with later‐onset diabetes might hinder the occurrence of cancer. Moreover, compared to patients with later‐onset diabetes, those with earlier‐onset diabetes received less optimal treatment and had poorer metabolic control in an Asian diabetic population^31^. Those with earlier‐onset diabetes were also found to be less likely to receive cancer prevention treatment with aspirin^32^, though they were more likely than those with later‐onset diabetes to receive sulfonylurea, which causes increased susceptibility to cancer (Table S3)^33^.

The causal relationship between diabetes and cancer might exist in both directions, in which an occult or existing cancer could induce metabolic derangements as part of paraneoplastic syndrome or in a form of reverse causality in pancreatic cancer^34^. Approximately 8–18% of patients with cancer have already been diagnosed with diabetes at the time of their cancer diagnosis^35^. As the highest cancer risk among patients with newly diagnosed diabetes occurs within the first year, at least a one‐year latency period is required for causal effects on cancer etiology^36^. The similar cancer risks with lag times of 1, 2, or 3 years support the association between the severity of diabetic complications and incident cancer in a temporal order.

Our study had several strengths. First, we included a large sample size of 616,742 patients with diabetes recorded over the course of more than 5 million person‐years at risk. Second, the comparability of the characteristics of the study participants and clinical significance was high because we exclusively enrolled patients with diabetes. Third, our study specifically evaluated the association between newly diagnosed diabetes and incident cancer and prevalent cohort bias might be avoided by excluding survivors of early incident diabetes who have a longer diabetic duration and higher susceptibility to cancer. Our study also had a few limitations, such as a lack of information regarding body mass index, laboratory results, and smoking and alcohol consumption habits. Further studies should investigate the relationship between the severity of diabetic complications, cancer, and adiposity in diabetic patients.

Given the observed increased risk of cancer associated with more severe diabetic complications, especially among those with earlier‐onset diabetes, enhanced cancer screening protocols should be considered. Such screenings should be targeted towards high‐risk groups identified using the aDCSI, thereby improving early detection and prevention strategies. In summary, the increased risk of cancer among patients with greater severity of diabetic complications suggests recommending enhanced cancer screening beyond that offered to those with less severe diabetes, particularly in those with earlier‐onset diabetes. The development of risk assessment tools for cancer using the aDCSI in patients with diabetes may facilitate the recognition of at‐risk populations and promote early cancer prevention.

## FUNDING

This study was financially supported by Tungs' Taichung Metroharbor Hospital, Taiwan (grant number TTMHH‐R1120076), the National Science and Technology Council (NSTC‐112‐2621‐M‐002‐024‐MY3), Taiwan, and the National Health Research Institutes (NHRI‐EM‐113‐GP‐02, NHRI‐EM‐113‐GP‐03, and NHRI‐EM‐113‐PP‐01), Taiwan. All authors have completed and submitted the ICMJE Form for Disclosure of Potential Conflicts of Interest and declare no other conflicts of interest. None of the funders had a role in the study's design, data collection, management, analysis, or interpretation, the manuscript's preparation, review, or approval, or the decision to submit the manuscript for publication.

## DISCLOSURE

The authors declare no conflict of interest.

Approval of the research protocol: Approved by the ethics review board of the National Taiwan University College of Public Health.

Informed consent: N/A.

Approval date of registry and the registration no. of the study/trial: N/A.

Animal studies: N/A.

## Supporting information


**Appendix S1.** Adapted Diabetes Complications Index and List of Complications Developed From *ICD‐9‐CM* Codes.
**Appendix S2.** Diagram of conceptual framework.
**Table S1.** Univariate and Multivariate ORs for cancer according to the adapted Diabetes Complications Severity Index in quartiles by nested case‐control study analysis^†^.
**Table S2.** Age‐adjusted HRs for cancers in men according to the adapted Diabetes Complications Severity Index in quartiles.
**Table S3.** Baseline characteristics of diabetic patients by 5‐year age groups.
**Figure S1.** Adjusted hazard ratios for cancers from any cause according to the aDCSI and based on duration of diabetes.
**Figure S2.** Adjusted hazard ratios for cancer from any cause according to the aDCSI and based on lag time between the onset of diabetes and the development of cancer.
