# Supplementary material for: Association between severity of diabetic complications and risk of cancer in middle‐aged patients with type 2 diabetes
Source: J Diabetes Investig. 2024 Nov 22;16(1):16–24. doi: 10.1111/jdi.14364 (PMC11693530; doi:10.1111/jdi.14364)
Supplement: Supplementary file 1 — Appendix S1. Adapted Diabetes Complications Index and List of Complications Developed From ICD‐9‐CM Codes. Appendix S2. Diagram of conceptual framework. Table S1. Univariate and Multivariate ORs for cancer according to the adapted Diabetes Complications Severity Index in quartiles by nested case‐control study analysis†. Table S2. Age‐adjusted HRs for cancers in men according to the adapted Diabetes Complications Severity Index in quartiles. Table S3. Baseline characteristics of diabetic patients by 5‐year age groups. Figure S1. Adjusted hazard ratios for cancers from any cause according to the aDCSI and based on duration of diabetes. Figure S2. Adjusted hazard ratios for cancer from any cause according to the aDCSI and based on lag time between the onset of diabetes and the development of cancer. [file JDI-16-16-s001.docx]

**Association between Severity of Diabetes and Risk of Cancer in Middle-Aged Patients with Type 2 Diabetes**

**Online-Only Supplemental Material**

**eAppendix A.** Adapted Diabetes Complications Index and List of Complications Developed From *ICD-9-CM* Codes

| **Complication and *ICD-9-CM* Diagnosis** | ***ICD-9-CM* Code** | **aDCSI Score** | |
| --- | --- | --- | --- |
|  |  | 1 | 2 |
| **Retinopathy** |  |  |  |
| Diabetic ophthalmologic disease | 250.5x | • |  |
| Background retinopathy | 362.01 | • |  |
| Other retinopathy | 362.1 | • |  |
| Retinal edema | 362.83 | • |  |
| CSME | 362.53 | • |  |
| Other retinal disorders | 362.81, 362.82 | • |  |
| Proliferative retinopathy | 362.02 |  | •• |
| Retinal detachment | 361.xx |  | •• |
| Blindness | 369.xx .00-.99 |  | •• |
| Vitreous hemorrhage | 379.23 |  | •• |
| **Nephropathy** |  |  |  |
| Diabetic nephropathy | 250.4 | • |  |
| Acute glomerulonephritis | 580 | • |  |
| Nephrotic syndrome | 581 | • |  |
| Hypertension, nephrosis | 581.81 | • |  |
| Chronic glomerulonephritis | 582 | • |  |
| Nephritis/nephropathy | 583 | • |  |
| Chronic renal failure | 585 |  | •• |
| Renal failure NOS | 586 |  | •• |
| Renal insufficiency | 593.9 |  | •• |
| **Neuropathy** |  |  |  |
| Diabetic neuropathy | 356.9, 250.6 | • |  |
| Amyotrophy | 358.1 | • |  |
| Cranial nerve palsy | 951.0, 951.1, 951.3 | • |  |
| Mononeuropathy | 354.0-355.9 | • |  |
| Charcot’s arthropathy | 713.5 | • |  |
| Polyneuropathy | 357.2 | • |  |

**eAppendix A.** Adapted Diabetes Complications Index and List of Complications Developed From *ICD-9-CM* Codes (Continued)

| **Complication and *ICD-9-CM* Diagnosis** | ***ICD-9-CM* Code** | **aDCSI Score** | |
| --- | --- | --- | --- |
|  |  | 1 | 2 |
| **Cerebrovascular** |  |  |  |
| TIA | 435 | • |  |
| Stroke | 431, 433, 434, 436 |  | •• |
| **Cardiovascular** |  |  |  |
| Atherosclerosis | 440.xx | • |  |
| Other IHD | 411 | • |  |
| Angina pectoris | 413 | • |  |
| Other chronic IHD | 414 | • |  |
| Myocardial infarction | 410 |  | •• |
| Ventricular fibrillation, arrest | 427.1, 427.3 |  | •• |
| Atrial fibrillation, arrest | 427.4, 427.5 |  | •• |
| Other ASCVD | 429.2 | • |  |
| Old myocardial infarction | 412 |  | •• |
| Heart failure | 428 |  | •• |
| Atherosclerosis, severe | 440.23, 440.24 |  | •• |
| Aortic aneurysm/dissection | 441 |  | •• |
| **Peripheral vascular disease** |  |  |  |
| Diabetic PVD | 250.7 | • |  |
| Other aneurysm, LE | 442.3 | • |  |
| PVD | 443.81, 443.9 | • |  |
| Foot wound + complication | 892.1 | • |  |
| Claudication, intermittent | 443.9 | • |  |
| Embolism/thrombosis (LE) | 444.22 |  | •• |
| Gangrene | 785.4 |  | •• |
| Gas gangrene | 0.4 |  | •• |
| Ulcer of lower limbs | 707.1 |  | •• |

**eAppendix A.** Adapted Diabetes Complications Index and List of Complications Developed From *ICD-9-CM* Codes (Continued)

| **Complication and *ICD-9-CM* Diagnosis** | ***ICD-9-CM* Code** | **aDCSI Score** | |
| --- | --- | --- | --- |
|  |  | 1 | 2 |
| **Metabolic** |  |  |  |
| Ketoacidosis | 250.1 |  | •• |
| Hyperosmolar | 250.2 |  | •• |
| Other coma | 250.3 |  | •• |

Severity index was calculated and summed based on scale score on each complication, in which the 0 = no abnormality, 1 = some abnormality, 2 = severe abnormality. The one solid circle sign (•) denote that a count of 1 added to aDCSI and double solid circle sign (••) denote that a count of 2 added to aDCSI.

aDCSI, adapted Diabetes Complications Severity Index; ASCVD, atherosclerotic cardiovascular disease; CSME, cystoid macular edema/degeneration; DCSI, Diabetes Complications Severity Index; IHD, ischemic heart disease; ICD-9-CM, International Classification of Diseases, Ninth Revision, Clinical Modification; LE, lower extremity; NOS, not otherwise specified; PVD, peripheral vascular disease; TIA, transient ischemic attack.

**eAppendix B. Diagram of conceptual framework**

**Cohort follow up from onset of diabetes End of Study**

|  |  |  |  |  |  |  |  |  |  |
| --- | --- | --- | --- | --- | --- | --- | --- | --- | --- |

0 1 2 3 4 5 6 7 8 9

(follow-up years)

Annually time-varying variables :

aDCSI; age; status of comorbidities; any treatment for diabetes, hypertension and hyperlipidemia.

Baseline time-fixed variables:

Gender, social-economic status

Study conceptual framework diagram illustrating a cohort study showing follow-up from onset of diabetes and end with cancer, death, or the end of the study, whichever came first, in which the black lines ending in a circles denote the event of cancer. The baseline time-fixed variables and subsequent annually time-varying variables were collected during follow-up.

**Table S1. Univariate and Multivariate ORs for cancer according to the adapted Diabetes Complications Severity Index in quartiles by nested case-control study analysis** †

| aDCSI | 0 | 1 | 2 | 3 | 4 | 5+ | P for trend |
| --- | --- | --- | --- | --- | --- | --- | --- |
| Men |  |  |  |  |  |  |  |
| Cases N (%) | 6,693 (25.8) | 3,341 (20.8) | 2,818 (20.6) | 1,448 (18.0) | 941 (16.7) | 1,105 (14.6) |  |
| Controls N (%) | 19,269 (74.2) | 12,751 (79.2) | 10,897 (79.5) | 6,585 (82.0) | 4,693 (83.3) | 6,461 (85.4) |  |
| Univariate OR  (95% CI) | 1.00 | 0.99 (0.95-1.04) | 1.10 (1.05-1.16) | 1.18 (1.10-1.26) | 1.18 (1.09-1.28) | 1.42 (1.30-1.54) | <0.001 |
| Multivariate OR †  (95% CI) | 1.00 | 1.00 (0.95-1.05) | 1.11 (1.05-1.18) | 1.17 (1.09-1.26) | 1.17 (1.07-1.28) | 1.39 (1.27-1.52) | <0.001 |
| Women |  |  |  |  |  |  |  |
| Cases N (%) | 3,794 (25.2) | 2,463 (21.2) | 1,836 (20.2) | 957 (18.0) | 569 (16.4) | 639 (14.6) |  |
| Controls N (%) | 11,281 (74.8) | 9,140 (78.8) | 7,261 (79.8) | 4,369 (82.0) | 2,904 (83.6) | 3,736 (85.4) |  |
| Univariate OR  (95% CI) | 1.00 | 1.05 (1.00-1.12) | 1.17 (1.10-1.25) | 1.22 (1.12-1.33) | 1.29 (1.15-1.43) | 1.57 (1.41-1.76) | <0.001 |
| Multivariate OR †  (95% CI) | 1.00 | 1.04 (0.98-1.11) | 1.14 (1.06-1.22) | 1.15 (1.06-1.26) | 1.22 (1.09-1.37) | 1.52 (1.35-1.70) | <0.001 |

Abbreviations: aDCSI, adapted Diabetes Complications Severity Index; CI, confidence interval; OR, odds ratio

† Nested case-control analysis using the same data of cohort with incidence density sampling at 1:4 ratio and match on the covariates of gender, status of income and urbanization and age (± 2 years)

‡ Adjusted for status of hypertension, hyperlipidemia, medication for hyperglycemia, hypertension, and hyperlipidemia, and use of aspirin

**Table S2. Age-adjusted HRs for cancers in men according to the adapted Diabetes Complications Severity Index in quartiles**

|  | 0 | 1 | 2 | 3 | 4 | 5 | P for trend |
| --- | --- | --- | --- | --- | --- | --- | --- |
| Men |  |  |  |  |  |  |  |
| Prostate |  |  |  |  |  |  |  |
| No. cases | 234 | 148 | 141 | 83 | 50 | 55 |  |
| Incidence | 18.1 | 23.1 | 28.8 | 34.1 | 32.2 | 30.5 |  |
| Adjusted HR †  (95% CI) | 1.00 | 1.08 (0.88-1.33) | 1.27 (1.02-1.56) | 1.38 (1.07-1.78) | 1.26 (0.93-1.72) | 1.16 (0.85-1.57) | 0.042 |
| Colorectal |  |  |  |  |  |  |  |
| No. cases | 825 | 483 | 417 | 241 | 159 | 225 |  |
| Incidence | 63.8 | 75.4 | 85.3 | 99.0 | 102.5 | 124.6 |  |
| Adjusted HR †  (95% CI) | 1.00 | 1.03 (0.92-1.16) | 1.11 (0.99-1.25) | 1.22 (1.05-1.41) | 1.23 (1.04-1.46) | 1.48 (1.28-1.72) | <.001 |
| Pancreas |  |  |  |  |  |  |  |
| No. cases | 159 | 95 | 68 | 35 | 33 | 36 |  |
| Incidence | 12.3 | 14.8 | 13.9 | 14.4 | 21.3 | 19.9 |  |
| Adjusted HR †  (95% CI) | 1.00 | 1.15 (0.89-1.48) | 1.04 (0.78-1.39) | 1.07 (0.74-1.55) | 1.58 (1.08-2.31) | 1.57 (1.09-2.27) | 0.006 |
| Hepatocellular |  |  |  |  |  |  |  |
| No. cases | 2282 | 1143 | 1043 | 495 | 343 | 430 |  |
| Incidence | 176.4 | 178.5 | 213.3 | 203.4 | 221.2 | 238.1 |  |
| Adjusted HR †  (95% CI) | 1.00 | 0.90 (0.84-0.97) | 1.03 (0.96-1.11) | 0.94 (0.85-1.04) | 1.01 (0.9-1.13) | 1.10 (0.99-1.22) | 0.06 |
| Lung |  |  |  |  |  |  |  |
| No. cases | 497 | 304 | 263 | 152 | 125 | 138 |  |
| Incidence | 38.4 | 47.5 | 53.8 | 62.5 | 80.6 | 76.4 |  |
| Adjusted HR †  (95% CI) | 1.00 | 1.09 (0.94-1.26) | 1.18 (1.01-1.37) | 1.29 (1.08-1.55) | 1.63 (1.34-1.99) | 1.55 (1.27-1.89) | <.001 |
| Urinary tract |  |  |  |  |  |  |  |
| No. cases | 30 | 22 | 28 | 16 | 13 | 10 |  |
| Incidence | 2.3 | 3.4 | 5.7 | 6.6 | 8.4 | 5.5 |  |
| Adjusted HR †  (95% CI) | 1.00 | 1.25 (0.72-2.19) | 1.96 (1.16-3.32) | 2.07 (1.11-3.87) | 2.56 (1.32-4.96) | 1.63 (0.79-3.38) | 0.002 |
| Kidney |  |  |  |  |  |  |  |
| No. cases | 38 | 22 | 23 | 21 | 9 | 23 |  |
| Incidence | 2.9 | 3.4 | 4.7 | 8.6 | 5.8 | 12.7 |  |
| Adjusted HR †  (95% CI) | 1.00 | 1.05 (0.62-1.77) | 1.37 (0.81-2.33) | 2.39 (1.4-4.08) | 1.56 (0.75-3.25) | 3.38 (2.02-5.65) | <.001 |
| Bladder |  |  |  |  |  |  |  |
| No. cases | 180 | 100 | 107 | 51 | 43 | 66 |  |
| Incidence | 13.9 | 15.6 | 21.9 | 21.0 | 27.7 | 36.6 |  |
| Adjusted HR †  (95% CI) | 1.00 | 1 (0.78-1.28) | 1.35 (1.06-1.71) | 1.23 (0.90-1.69) | 1.6 (1.15-2.25) | 2.15 (1.59-2.89) | <.001 |
| Stomach |  |  |  |  |  |  |  |
| No. cases | 218 | 102 | 130 | 92 | 40 | 51 |  |
| Incidence | 16.9 | 15.9 | 26.6 | 37.8 | 25.8 | 28.2 |  |
| Adjusted HR †  (95% CI) | 1.00 | 0.84 (0.66-1.06) | 1.33 (1.07-1.66) | 1.8 (1.4-2.31) | 1.2 (0.85-1.69) | 1.32 (0.97-1.8) | <.001 |
| Lymphoma |  |  |  |  |  |  |  |
| No. cases | 27 | 20 | 20 | 9 | 11 | 4 |  |
| Incidence | 2.1 | 3.1 | 4.1 | 3.7 | 7.1 | 2.2 |  |
| Adjusted HR †  (95% CI) | 1.00 | 1.27 (0.71-2.27) | 1.56 (0.88-2.76) | 1.3 (0.60-2.81) | 2.41 (1.17-4.98) | 0.73 (0.26-2.07) | 0.462 |
| Leukemia |  |  |  |  |  |  |  |
| No. cases | 57 | 32 | 30 | 22 | 15 | 16 |  |
| Incidence | 4.4 | 5.0 | 6.1 | 9.0 | 9.7 | 8.9 |  |
| Adjusted HR †  (95% CI) | 1.00 | 1.05 (0.68-1.61) | 1.24 (0.79-1.95) | 1.79 (1.08-2.97) | 1.91 (1.06-3.44) | 1.84 (1.06-3.19) | <.001 |

Abbreviations: CI, confidence interval; HR, hazard ratio.

† Adjusted for age, urbanization, and income

**Table S2. Age-adjusted HRs for cancers in women according to the Diabetes Complications Severity Index in quartiles (continued)**

|  | 0 | 1 | 2 | 3 | 4 | 5 | P for trend |
| --- | --- | --- | --- | --- | --- | --- | --- |
| Women |  |  |  |  |  |  |  |
| Premenopausal breast |  |  |  |  |  |  |  |
| No. cases | 272 | 165 | 120 | 26 | 28 | 27 |  |
| Incidence | 79.6 | 89.9 | 110.6 | 48.8 | 95.9 | 82.0 |  |
| Adjusted HR †  (95% CI) | 1.00 | 1.04 (0.86-1.26) | 1.28 (1.03-1.59) | 0.57 (0.38-0.85) | 1.14 (0.77-1.69) | 1.03 (0.69-1.53) | 0.945 |
| Postmenopausal breast |  |  |  |  |  |  |  |
| No. cases | 591 | 396 | 287 | 166 | 87 | 72 |  |
| Incidence | 94 | 92.5 | 94.1 | 99.9 | 89.8 | 70.6 |  |
| Adjusted HR †  (95% CI) | 1.00 | 0.91 (0.8-1.04) | 0.91 (0.79-1.05) | 0.97 (0.81-1.15) | 0.87 (0.7-1.1) | 0.73 (0.57-0.94) | 0.031 |
| Ovarian |  |  |  |  |  |  |  |
| No. cases | 92 | 70 | 42 | 21 | 16 | 8 |  |
| Incidence | 9.5 | 11.4 | 10.2 | 9.6 | 12.7 | 5.9 |  |
| Adjusted HR †  (95% CI) | 1.00 | 1.12 (0.82-1.53) | 0.97 (0.67-1.4) | 0.92 (0.57-1.49) | 1.24 (0.72-2.13) | 0.63 (0.31-1.29) | 0.662 |
| Endometrial |  |  |  |  |  |  |  |
| No. cases | 212 | 118 | 82 | 43 | 25 | 27 |  |
| Incidence | 21.8 | 19.3 | 19.8 | 19.6 | 19.8 | 20.0 |  |
| Adjusted HR †  (95% CI) | 1.00 | 0.82 (0.65-1.03) | 0.83 (0.64-1.07) | 0.82 (0.59-1.14) | 0.84 (0.55-1.27) | 0.92 (0.61-1.38) | 0.413 |
| Colorectal |  |  |  |  |  |  |  |
| No. cases | 466 | 367 | 246 | 154 | 98 | 120 |  |
| Incidence | 48 | 60 | 59.5 | 70.2 | 77.7 | 89.0 |  |
| Adjusted HR †  (95% CI) | 1.00 | 1.11 (0.96-1.27) | 1.05 (0.90-1.23) | 1.2 (1.00-1.44) | 1.32 (1.06-1.65) | 1.58 (1.28-1.94) | <.001 |
| Pancreas |  |  |  |  |  |  |  |
| No. cases | 77 | 53 | 45 | 26 | 11 | 13 |  |
| Incidence | 7.9 | 8.7 | 10.9 | 11.8 | 8.7 | 9.6 |  |
| Adjusted HR †  (95% CI) | 1.00 | 0.96 (0.68-1.36) | 1.14 (0.79-1.66) | 1.2 (0.76-1.87) | 0.87 (0.46-1.64) | 0.98 (0.54-1.77) | 0.917 |
| Hepatocellular |  |  |  |  |  |  |  |
| No. cases | 590 | 436 | 373 | 241 | 128 | 183 |  |
| Incidence | 60.8 | 71.3 | 90.2 | 109.8 | 101.5 | 135.7 |  |
| Adjusted HR †  (95% CI) | 1.00 | 1.00 (0.88-1.13) | 1.19 (1.04-1.36) | 1.38 (1.18-1.60) | 1.24 (1.02-1.51) | 1.69 (1.43-2.00) | <.001 |
| Lung |  |  |  |  |  |  |  |
| No. cases | 240 | 178 | 166 | 84 | 50 | 56 |  |
| Incidence | 24.7 | 29.1 | 40.2 | 38.3 | 39.7 | 41.5 |  |
| Adjusted HR †  (95% CI) | 1.00 | 1.01 (0.83-1.23) | 1.32 (1.07-1.61) | 1.19 (0.93-1.54) | 1.21 (0.89-1.65) | 1.28 (0.95-1.73) | 0.120 |
| Urinary tract |  |  |  |  |  |  |  |
| No. cases | 24 | 31 | 49 | 16 | 18 | 25 |  |
| Incidence | 2.5 | 5.1 | 11.9 | 7.3 | 14.3 | 18.5 |  |
| Adjusted HR †  (95% CI) | 1.00 | 1.75 (1.02-2.98) | 3.82 (2.32-6.31) | 2.23 (1.17-4.24) | 4.26 (2.26-8.05) | 5.62 (3.11-10.17) | <.001 |
| Kidney |  |  |  |  |  |  |  |
| No. cases | 17 | 10 | 11 | 15 | 7 | 11 |  |
| Incidence | 1.8 | 1.6 | 2.7 | 6.8 | 5.6 | 8.2 |  |
| Adjusted HR †  (95% CI) | 1.00 | 0.81 (0.38-1.74) | 1.25 (0.58-2.66) | 3.07 (1.55-6.09) | 2.45 (0.98-6.16) | 3.72 (1.76-7.88) | <.001 |
| Bladder |  |  |  |  |  |  |  |
| No. cases | 63 | 57 | 49 | 27 | 17 | 38 |  |
| Incidence | 6.5 | 9.3 | 11.9 | 12.3 | 13.5 | 28.2 |  |
| Adjusted HR †  (95% CI) | 1.00 | 1.26 (0.88-1.8) | 1.54 (1.05-2.24) | 1.55 (0.98-2.45) | 1.7 (0.98-2.92) | 3.72 (2.43-5.69) | <.001 |
| Stomach |  |  |  |  |  |  |  |
| No. cases | 126 | 69 | 60 | 33 | 15 | 32 |  |
| Incidence | 13.0 | 11.3 | 14.5 | 15.0 | 11.9 | 23.7 |  |
| Adjusted HR †  (95% CI) | 1.00 | 0.76 (0.57-1.03) | 0.94 (0.69-1.28) | 0.95 (0.64-1.4) | 0.75 (0.43-1.29) | 1.57 (1.05-2.34) | 0.110 |
| Lymphoma |  |  |  |  |  |  |  |
| No. cases | 25 | 12 | 16 | 10 | 2 | 2 |  |
| Incidence | 2.6 | 2 | 3.9 | 4.6 | 1.6 | 1.5 |  |
| Adjusted HR †  (95% CI) | 1.00 | 0.68 (0.34-1.33) | 1.29 (0.69-2.41) | 1.5 (0.72-3.12) | 0.53 (0.12-2.25) | 0.53 (0.12-2.27) | 0.680 |
| Leukemia |  |  |  |  |  |  |  |
| No. cases | 37 | 36 | 34 | 11 | 8 | 13 |  |
| Incidence | 3.8 | 5.9 | 8.2 | 5.0 | 6.3 | 9.6 |  |
| Adjusted HR †  (95% CI) | 1.00 | 1.4 (0.88-2.24) | 1.88 (1.16-3.05) | 1.12 (0.56-2.24) | 1.41 (0.65-3.07) | 2.25 (1.16-4.35) | 0.024 |

Abbreviations: CI, confidence interval; HR, hazard ratio.

† Adjusted for age, urbanization, and income

**Table S3. Baseline characteristics of diabetic patients by 5-year age groups**

| Characteristics | 40-44  (n=86,569) | 45-49  (n=126,747) | 50-54  (n=149,463) | 55-59  (n=138,827) | 60-64  (n=115,136) |
| --- | --- | --- | --- | --- | --- |
| Age – years † | 42.6±1.4 | 47.6±1.4 | 52.5±1.4 | 57.4±1.4 | 62.4±1.4 |
| Women – No. (%) | 34.3 | 38.4 | 44.8 | 47.8 | 50.9 |
| Follow-up – years † | 9.3±3.8 | 9.2±3.8 | 8.8±3.7 | 8.5±3.8 | 8.8±3.8 |
| Income (NTD) |  |  |  |  |  |
| 0 | 8.2 | 8.5 | 9.5 | 10.9 | 14.4 |
| 1-15,840 | 20.1 | 16.3 | 13.7 | 11.7 | 10.6 |
| 15,840-25,000 | 45.8 | 48.2 | 49.4 | 50.3 | 51.8 |
| >25,000 | 26.0 | 27.0 | 27.4 | 27.1 | 23.2 |
| Urbanization |  |  |  |  |  |
| Low | 45.5 | 44.3 | 43.8 | 42.7 | 40.4 |
| Moderate | 40.9 | 41.5 | 41.3 | 40.8 | 39.4 |
| High | 9.4 | 10.0 | 10.2 | 11.0 | 13.0 |
| Very high | 4.1 | 4.2 | 4.7 | 5.5 | 7.1 |
| Medical diseases |  |  |  |  |  |
| Hypertension | 15.5 | 20.6 | 27.3 | 32.6 | 36.7 |
| Hyperlipidemia | 12.9 | 14.2 | 16.8 | 19.3 | 19.8 |
| Medication |  |  |  |  |  |
| Metformin | 2.9 | 3.2 | 3.5 | 4.2 | 5.0 |
| Sulfonylurea | 6.4 | 7.0 | 7.4 | 8.7 | 10.6 |
| Acarbose | 0.2 | 0.2 | 0.2 | 0.3 | 0.4 |
| TZD | 0.3 | 0.3 | 0.3 | 0.4 | 0.4 |
| DPP4 inhibitor | 0.1 | <0.1 | <0.1 | 0.1 | 0.1 |
| Insulin | 1.0 | 0.9 | 0.8 | 0.8 | 1.1 |
| CCB | 1.7 | 2.2 | 2.8 | 3.3 | 3.9 |
| Beta-blocker | 4.5 | 5.9 | 7.2 | 8.0 | 9.0 |
| ACEI | 5.4 | 7.2 | 8.8 | 10.3 | 12.5 |
| ARB | 3.7 | 4.8 | 6.4 | 7.8 | 8.8 |
| Aspirin | 3.2 | 5.0 | 7.4 | 9.9 | 12.6 |
| Statins | 2.7 | 3.6 | 4.9 | 6.4 | 6.8 |
| aDCSI | 0.20±0.60 | 0.26±0.68 | 0.35±0.79 | 0.45±0.9 | 0.56±1.01 |

Abbreviations: NTD, New Taiwan dollars; TZD, thiazolidinedione; DPP4, dipeptidyl peptidase 4; CCB, calcium channel blocker; ACEI, angiotensin-converting enzyme inhibitor; ARB, angiotensin II receptor blocker; aDCSI, adapted Diabetes Complications Severity Index

† Values represent means±SD.

**Figure S1. Adjusted hazard ratios for cancers from any cause according to the aDCSI and based on duration of diabetes**


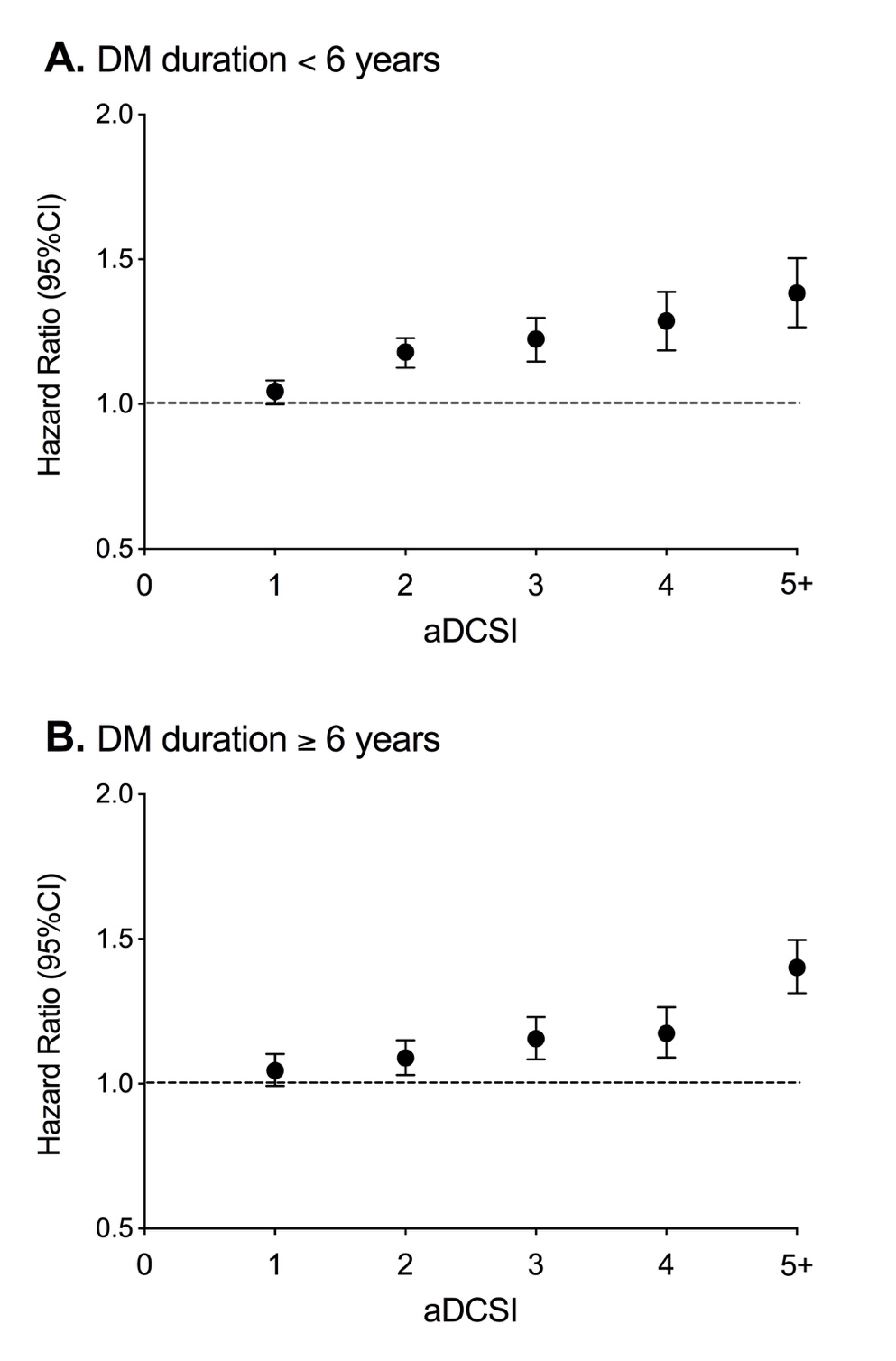


Adjusted hazard ratio for cancer from any cause according to the aDCSI from 0 to 5+, as adjusted for age, sex and socioeconomic status with accounting for the competing risk of death. (A) Duration of diabetes <6 years; (B) duration of diabetes ≥6 years; aDCSI=adapted Diabetes Complication Severity Index; CI=confidence interval.

**Figure S2. Adjusted hazard ratios for cancer from any cause according to the aDCSI and based on lag time between the onset of diabetes and the development of cancer**


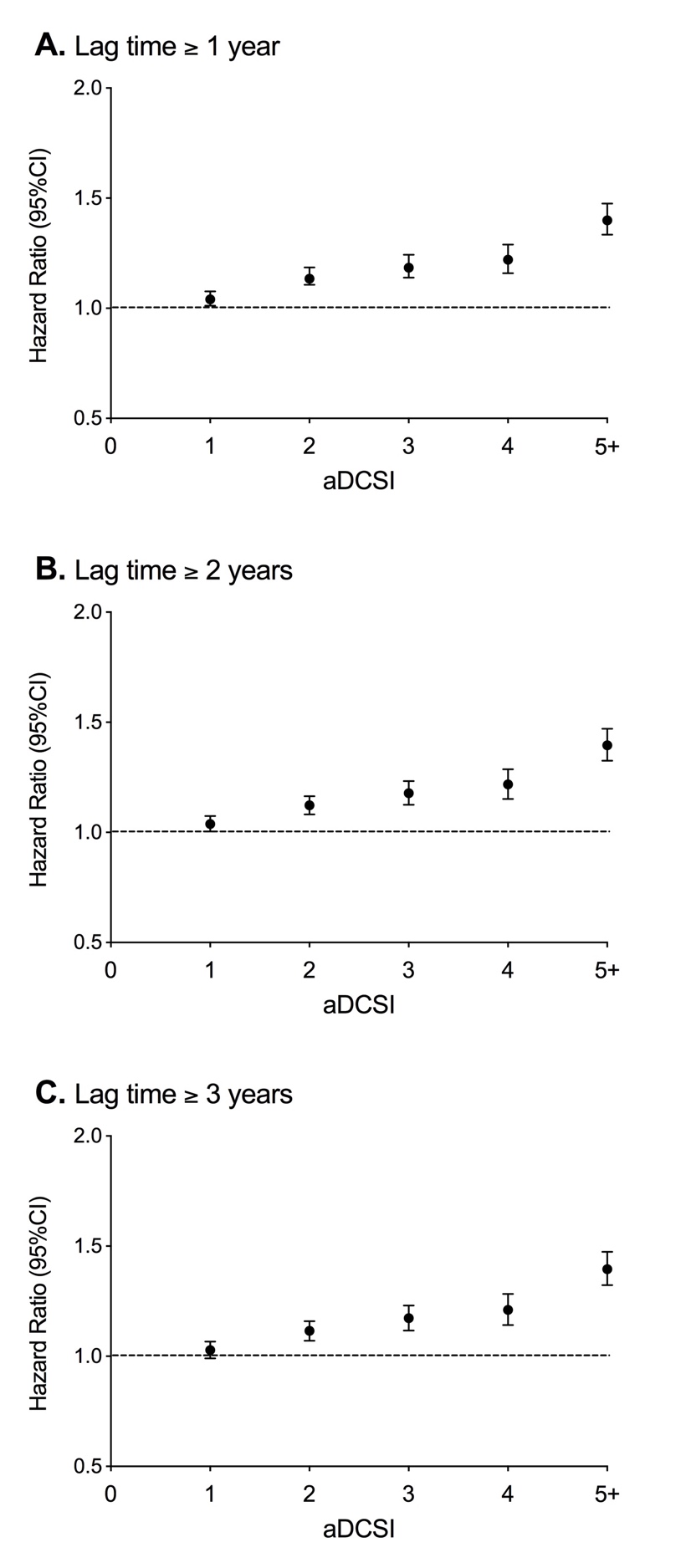


Hazard ratios for cancer from any cause according to the aDCSI from 0 to 5+, as adjusted for age, sex and socioeconomic status accounting for the competing risk of death. (A) Lag time ≥1 year; (B) lag time ≥2 years; (C) lag time ≥3 years; aDCSI=adapted Diabetes Complication Severity Index; CI=confidence interval
